# Thermodynamics of Observations

**DOI:** 10.3390/e27090968

**Published:** 2025-09-17

**Authors:** Arno Keppens, Jean-Christopher Lambert

**Affiliations:** Royal Belgian Institute for Space Aeronomy (BIRA-IASB), 1180 Brussels, Belgium

**Keywords:** classical thermodynamics, observation distributions, partition function, statistical ensembles, uncertainty assessment

## Abstract

This work demonstrates that the four laws of classical thermodynamics apply to the statistics of symmetric observation distributions, and provides examples of how this can be exploited in uncertainty assessments. First, an expression for the partition function *Z* is derived. In contrast with general classical thermodynamics, however, this can be performed without the need for variational calculus, while *Z* also equals the number of observations *N* directly. Apart from the partition function Z≡N as a scaling factor, three state variables *m*, *n*, and ϵ fully statistically characterize the observation distribution, corresponding to its expectation value, degrees of freedom, and random error, respectively. Each term in the first law of thermodynamics is then shown to be a variation on $\delta m^{2}=\delta {\left(n\epsilon \right)}^{2}$ for both canonical (constant *n* and ϵ) and macro-canonical (constant ϵ) observation ensembles, while micro-canonical ensembles correspond to a single observation result bin having $\delta m^{2}=0$. This view enables the improved fitting and combining of observation distributions, capturing both measurand variability and measurement precision.

## 1. Introduction

The laws of thermodynamics outline the constraints that systems obey in their statistical behavior, usually in terms of state variables and for (quasi) equilibrium states. They can be summarized as (T0) the transitivity of thermal equilibrium; (T1) energy conservation; (T2) entropy increase with time; and (T3) non-zero temperature. The universality of these laws has surprised for nearly two centuries, since the dawn of thermodynamics as a discipline, and is related to what one can possibly know about a system near thermal equilibrium [1]. It is typically irrelevant, however, how this thermodynamic information is obtained. In contrast therewith, in this work we explicitly assert that this information, and the thermodynamic assessment based thereon, results from registered perceptions (whether fictive or real) of a predefined measurand *m* that is subject to noise. This noise component can originate from random variations in the measurand, called variability, or from random errors in the measurement system, called precision. Usually, in practice, both appear combinedly. This does not affect the analysis below, although it is easier to consider either one or the other in order not to mix the degrees of freedom of the observed and observing systems (also see the section on demonstrative applications). In any case, our goal is a thermodynamic assessment resulting from an ensemble of observations, meaning that its statistics is captured by the observation distribution’s state variables and the laws of thermodynamics connecting them.

The distribution of repeated discrete observations is typically approximated by continuous Gaussian statistics. A connection with the arguably more appropriate equibinomial distribution, which is discrete by construction but has the normal distribution as its continuum limit (cf. de Moivre–Laplace theorem), is missing. This work starts with the derivation of a thermodynamic partition function from an equibinomial observation distribution, without the usual need for variational calculus [2]. A discussion on the statistical (observation) ensembles that relate with this partition function follows next, and enables a tailored reformulation of the four laws of thermodynamics. Before conclusions are drawn, five demonstrative applications put this work into experimental context. These touch upon, but are not limited to, uncertainty assessment discussions within the atmospheric research community that initiated this work [3,4].

## 2. Partition Function

**Figure 1:**
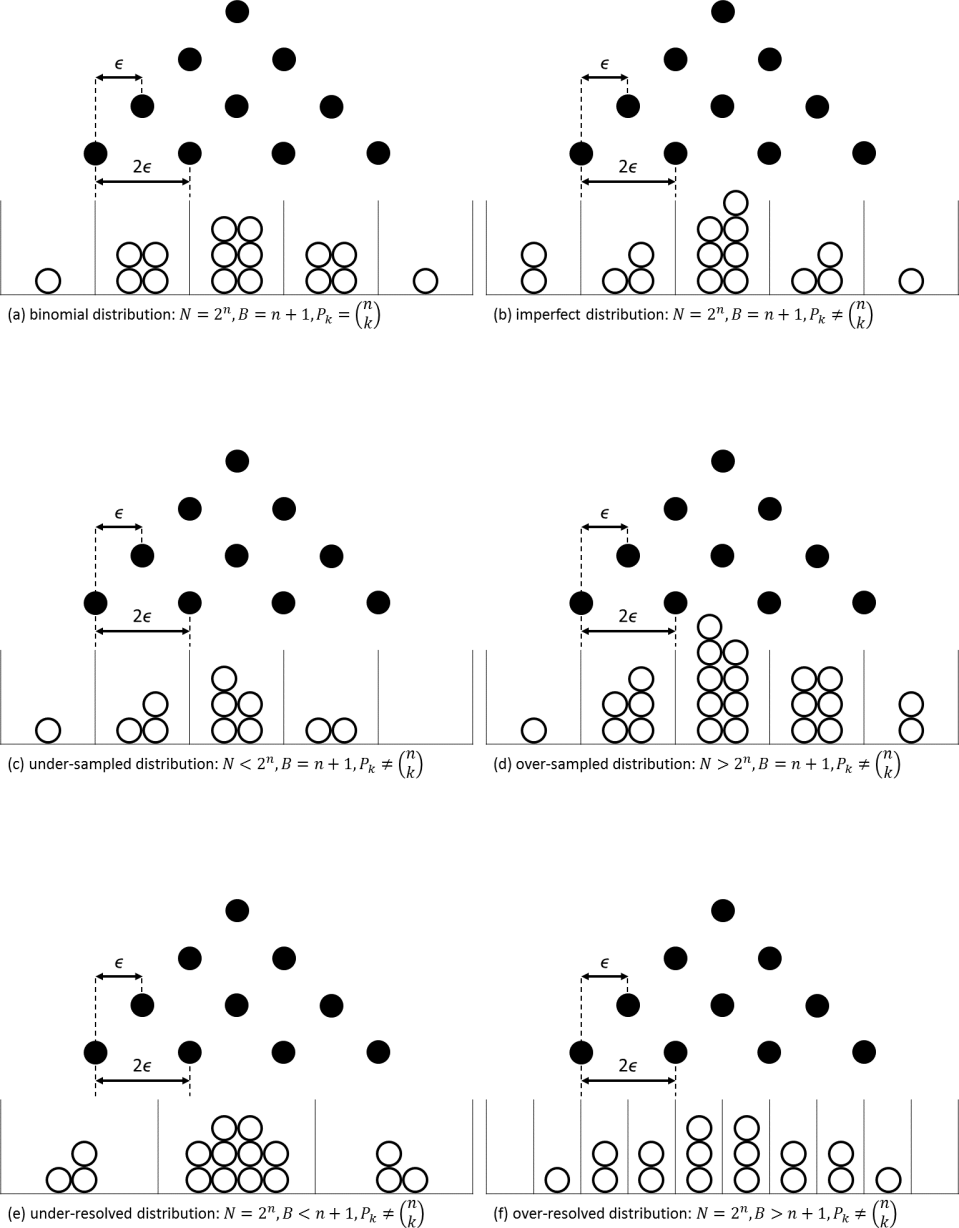
Variations on the equibinomial distribution $P_{k}$ upon fitting *N* observations (white circles) into *B* result bins, for a Galton board (black circles) with n=4 levels and noise error ϵ.

The trajectory of a single ball on a Galton board with *n* levels is essentially a sequence of *n* left–right choices. It can be described by a Bernoulli process consisting of *n* trials. A single observation of a measurand *m* can be identified with a Bernoulli process analogously, with each of its *n* degrees of freedom contributing a noise component +ϵ or −ϵ to the measurand *m* fully randomly, i.e., with equal probability p=0.5 (irrespective of whether the noise originates from the observed system or the observing system). A sequence of *N* independent observations of *m* hence consists of Nn Bernoulli trials within *N* trajectories. Combinatorics dictates that $2^{n}$ unique trajectories can be followed, each with equal probability $1/2^{n}$, but some trajectories will yield the same result in agreement with the binomial coefficients for an unordered selection of *k* out of *n*: the observation outcomes are located at m+(2k−n)ϵ if *k* represents the number of positive noise contributions, irrespective of their order within the trajectory, with 0≤k≤n. This means that the *N* measured values of *m* are distributed over B=n+1 result bins of width 2ϵ, centered at *m*. They follow the equibinomial distribution by construction if $N=2^{n}$ and in the absence of statistical fluctuations, see Figure 1a, as assumed in the remainder of this section. Figure 1b provides an example of an imperfect distribution in the presence of statistical fluctuations. The distribution is under-sampled for $N<2^{n}$ (Figure 1c), while it is over-sampled for $N>2^{n}$ (Figure 1d, also see next section). On the other hand, if the number of result bins can be chosen at will, the distribution can be called under-resolved for B<n+1 (Figure 1e) and over-resolved for B>n+1 (Figure 1f), respectively.

The binomial probability density $p_{k}$ of bin *k* is given by

(1) pk=nkpk(1−p)n−k=nk2−n

where the last equation holds for the equibinomial distribution with p=0.5. This means that for $N=2^{n}$ observations, the number of results per bin $P_{k}$ equals the binomial coefficient:

(2) Pk=nk=n!k!(n−k)!

This is demonstrated in Figure 1 for n=4. The sum over all states $P_{k}$ is called the partition function *Z* (from *Zustandssumme* in German), which here is fully characterized:

(3) Z=∑k=0nPk=∑k=0nnk=∑k=0n2npk=2n

The equibinomial distribution corresponds to a discrete probability density function $p_{k}$ that, according to the de Moivre–Laplace theorem, approaches the continuous probability density function $p_{x}$ of the normal distribution ϕ(x,μ,σ) for large *n* (or *N*, and possibly given a continuity correction):

(4) $$p_{k}\approx p_{x_{k}\pm \epsilon }=\int_{x_{k}-\epsilon }^{x_{k}+\epsilon }\phi \left(x,\mu ,\sigma \right)dx$$

for $x_{k}=m+\left(2k-n\right)\epsilon$ to match the *k* binomial bins of width 2ϵ, numbered 1 to n+1. Instead of using the Gauss error function to determine this definite integral, for sufficiently large *n*, it can be straightforwardly approximated by $2\epsilon \;\phi (x_{k},\mu ,\sigma )$, resulting in

(5) $$p_{k}\approx \frac{2\epsilon }{\sigma \sqrt{2\pi }}\exp\left[-\frac{{\left(x_{k}-\mu \right)}^{2}}{2\sigma^{2}}\right]$$

with $\sum_{k}p_{k}$ still approximately equal to one as required for a probability density. By insertion of the expression for $x_{k}$, the expectation (or mean) μ≡m, and the standard deviation $\sigma =2\epsilon \sqrt{np(1-p)}$ in units of the bin width, or $\sigma =\epsilon \sqrt{n}$ for p=0.5 here, one obtains for the partition function

(6) $$Z=\sum_{k=0}^{n}2^{n}p_{k}\approx \sum_{k=0}^{n}\frac{2^{n+1}}{\sqrt{2\pi n}}\exp\left[-\frac{{\left(2k\epsilon -n\epsilon \right)}^{2}}{2n\epsilon^{2}}\right]$$

As the normal distribution is the (continuous) distribution that maximizes the Shannon or Gibbs entropy for a specified mean and variance, this approach provides a succinct approximation to the classical and discrete canonical partition function for thermodynamic equilibrium states, without the need for variational calculus as in the Jaynesian maximum entropy approach [2]. In its general thermodynamic form, it is written as

(7) $$Z=\sum_{k}z_{k}\exp\left(-\beta E_{k}\right)$$

with $z_{k}$ accounting for the number of particles (or, here: observation trajectories $P_{k}$ yielding bin *k*) involved and $E_{k}$ the energy of state (or bin) *k*, to be determined later on. β equals the reciprocal of the Boltzmann constant times a temperature. Assuming a perfect equibinomial distribution, $z_{k}$ is uniquely determined by *n* (again see Figure 1 for deviations therefrom). In contrast with Equation (7), however, Equation (6) provides an exact formulation of the normalization factors and coefficients involved. It is therefore not surprising that the statistical “state variables” like (quadratic) mean, variance, and information content can be derived therefrom, fully analogous to the derivation of the energy *E*, temperature *T*, and entropy *S*, respectively, from the partition function in classical thermodynamics.

The relationships between the partition function and the thermodynamic variables of the system involve $\partial \log_{2}\left(Z\right)$, which straightforwardly equals ∂n for $Z\equiv N=2^{n}$ here, without having to go through the full differentiation of Equation (6). For the expectation value of the thermodynamic energy, one obtains

(8) $$\langle E\rangle =-\frac{\partial \log_{2}\left(Z\right)}{\partial \beta }=\frac{nk_{B}T}{2}={\left(n\epsilon \right)}^{2}$$

from $\beta =1/k_{B}T$, and upon identification of the exponential denominators of Equations (6) and (7), $k_{B}T\equiv 2n\epsilon^{2}$. This result confirms that $\langle E\rangle$ equals the quadratic mean of the observation distribution, apart from a constant offset. The temperature variable thus provides a measure of the energy per degree of freedom and of the observation ensemble variance, apart from half the Boltzmann constant $k_{B}$ as a unit conversion factor. The entropy of the distribution as determined from the partition function equals $\partial \left(k_{B}T\log_{2}Z\right)/\partial T=k_{B}n$ as a direct measure of the system’s degrees of freedom. The entropy as such is proportional to the base-two logarithm of the number of possible Bernoulli process outcomes indeed. In other words, the partition function provides a missing parameter, being that the equibinomial distribution integral is a scaling factor, for fully characterizing a distribution of observations in thermal equilibrium (also see next sections). In the absence of scaling, the latter still requires three state variables (*m*, *n*, ϵ) instead of two as for the normal distribution (μ, σ).

## 3. Statistical Ensembles

The partition function for equibinomial observation distributions presented in Equation (6) can easily be related to the well-known statistical ensembles of classical thermodynamics [2]: For a fixed number of observations $N≳2^{n}$ with result binning according to the equibinomial distribution, the canonical ensemble is obtained (see Table 1). The macro-canonical ensemble includes several canonical ensembles with the same random error ϵ but different numbers of degrees of freedom *n*. For N=1, one has one observation (bin) only, and hence the variance is undefined, in agreement with the fixed energy of the micro-canonical ensemble (again see Table 1). One however also obtains the micro-canonical ensemble, with finite entropy, upon forcing N>1 observations into a single (under-resolved, see Figure 1) result bin.

By assuming that within a single observation each of the *n* degrees of freedom of the system contributes a unique assessment of the measurand, each observation can also be regarded as a sequence of *n* discrete sub-observations (Bernoulli trials in the previous section). Therefore, in the common first law of thermodynamics, $\delta E=T\delta S+\mu_{E}\delta N_{E}$ (with subscripts *E* to distinguish from previous variables), the first term expresses that in a given distribution, each degree of freedom (sub-observation) comes with an energy (change) that is proportional to the variance $\sigma^{2}=k_{B}T/2$, or for each Bernoulli trial in Figure 1, *m* changes by ϵ. On the other hand, the second term expresses how the total energy (distribution) is affected by a change in the total number of degrees of freedom, which corresponds to varying the number of Bernoulli trials (rows) in Figure 1. However, for each degree of freedom added or removed, *m* again changes by +ϵ or −ϵ, respectively.

It hence becomes clear that each contribution to the first law of observation thermodynamics has to take the form $\delta m^{2}=\delta {\left(n\epsilon \right)}^{2}$ for both canonical (constant *n* and ϵ) and macro-canonical (constant ϵ) observation ensembles, while micro-canonical ensembles correspond to a single observation result bin having $\delta m^{2}=0$. For canonical ensembles with a fixed random error, one has $\delta m^{2}=\left(2n\epsilon^{2}\right)\delta n$, with $2n\epsilon^{2}$ representing the temperature as the double ensemble variance, while for a fixed number of degrees of freedom $\delta m^{2}=\left(2n^{2}\epsilon \right)\delta \epsilon$ still holds, capturing that the (quadratic) expectation value can vary due to a change in the system’s random error just as well.

## 4. Laws of Observation Thermodynamics

The above can be summarized into four laws of equibinomial observation statistics: (O1) An equibinomial observation distribution is fully characterized by its three state variables *m*, *n*, and ϵ, apart from an offset (additive bias) and scaling (*N*) that do not affect its equilibrium shape. (O2) The observation degrees of freedom *n* and random error per degree of freedom ϵ are positive numbers. (O3) Apart from a constant offset, an observation ensemble’s expectation value *m* is fully determined by *n* and ϵ: δm=δ(nϵ) and therefore $\delta m^{2}=\left(2n\epsilon^{2}\right)\delta n$ for constant ϵ. The double ensemble variance $2\sigma^{2}=2n\epsilon^{2}$ is also called its temperature. Micro-canonical ensembles correspond to a single observation result bin having $\delta m^{2}=0$. (O4) Every observation (ensemble) provides information that cannot be undone: δn≥0 (and hence δm≥0). This law essentially captures information conservation. It can also be expressed as ∂n/∂N≥0 or ∂n/∂t≥0, reflecting that subsequent observations (in time) cannot decrease the combined number of degrees of freedom. The equality only holds for the (over-)sampling (equivalent to scaling) of a given distribution.

This summary, however, does not match the laws of classical thermodynamics in their typical format. A rephrasing of the above laws in line with the known laws of thermodynamics may sound as follows: (T0) When two or more equibinomial observation distributions with the same $T=2n\epsilon^{2}$ are combined, the resulting distribution will (be over-sampled but still) have the same temperature. One may denote this as the transitivity of the variance, see Table 2. Note that this actually follows from O3. T0 therefore has no equivalent above. (T1) Apart from a constant offset, an ensemble’s expectation value *m* is fully determined by *n* and ϵ (again O3, as conservation of the expectation value). (T2) Information or entropy increase (cf. O4): Every observation (ensemble) provides information that cannot be undone, or ∂n/∂N≥0. For δn=0, no information is added and hence T0 is obtained (over-sampling). For $n_{+}=n_{1}+n_{2}$ (independent observations of the same measurand), one has $\sigma_{+}^{2}=\sigma_{1}^{2}+\sigma_{2}^{2}$. For correlated observations, $\sigma_{+}^{2}=\sigma_{1}^{2}+\sigma_{2}^{2}+2\rho \sigma_{1}\sigma_{2}$ with ρ being the correlation coefficient according to Gaussian statistics, meaning that the combined variance can go down (ρ<0 for anti-correlated observations) despite the increasing number of degrees of freedom. (T3) For any n>0, not all observations can yield exactly the same outcome (intrinsic random observation error or ϵ>0), from O2. This could be read as follows: as soon as there is a degree of freedom (or even a fraction thereof, see next section), you obtain an observation *distribution*, as you have to make a choice between going left or right by ϵ in the Bernoulli trajectory.

The laws of classical thermodynamics do not have a direct equivalent to O1, possibly because of it being just too obvious. It could be phrased as follows: A system in thermodynamic equilibrium is maximally statistically characterized by its state variables. Following Ginsberg’s theorem [5], O1 could say that there are just a few game pieces to play with; see the first row of Table 2. On the other hand, black hole mechanics, upon taking into account quantum-mechanical vacuum fluctuations near the black hole horizon, can be phrased in terms of the laws of thermodynamics as well [6] and does have an equivalent to O1. The latter is known as the no-hair theorem [7] but typically not related to black hole thermodynamics (BHT) as such in the literature. BHT makes a special case, however, as the relation between the three state variables mass *M*, surface gravity κ, and horizon area *A* is fixed at the Schwarzschild horizon, where the number of degrees of freedom is maximized ($N_{E}\equiv n$). This means *M* is exactly determined by κ and *A*, and not up to a constant: (4πG)M=κA, with $\kappa =c^{4}/4GM$, because $A=16\pi G^{2}M^{2}/c^{4}$ as well (from the Schwarzschild radius, with *G* as the gravitational constant and *c* as the speed of light). Actually, the laws of equilibrium thermodynamics apply more generally to all spherical surfaces that are concentric with the Schwarzschild horizon for Schwarzschild scenarios as rigorously proven in [8] and fully formulated in [9], taking into account that, hence, Bekenstein’s generalized second law of BHT needs to be adopted [10]. However, the energy that is confined within a spacetime region is only determined up to a constant, now in analogy with the first law of thermodynamics, for Schwarzschild–de Sitter scenarios with a cosmological constant, while the second term in T1 that accounts for a changing number of constituents can incorporate black hole evaporation by Hawking radiation.

**Table 2:** Comparison table for different versions of the laws of thermodynamics. The (+) denotes the possibility of additional terms. Italics in the first row are our own additions.

#	Observation Thermodynamics	Classical Thermodynamics [2]	Black Hole Thermodynamics [6]	Ginsberg’s Theorem [5]
-	An equibinomial observation distribution is maximally statistically characterized by its state variables (*m*, *n*, ϵ) and the number of observations *N*.	*A system in thermodynamic equilibrium is maximally statistically characterized by its state variables.*	No-hair theorem. ^1^	*There’s just a few pieces.*
T0	Transitivity of the variance: $\sigma_{n,\epsilon }=$ constant, irrespective of *N*.	Transitivity of thermal equilibrium: $T_{S}=$ constant.	The surface gravity of a black hole is constant over the event horizon: $\kappa_{A}=$ constant.	There is a game.
T1	$\delta m^{2}=\delta {\left(n\epsilon \right)}^{2}$	$\delta E=T\delta S+\mu_{E}\delta N_{E}$ (+)	δM=(κ/8π)δA (+)	You can’t win.
T2	If two observation distributions are combined, the joint d.o.f. at least equal the sum of the initial d.o.f.: $n_{+}\geq n_{1}+n_{2}$ or ∂n/∂t≥0.	If two thermodynamic systems are combined, the joint entropy at least equals the sum of the initial entropies: $S_{+}\geq S_{1}+S_{2}$ or ∂S/∂t≥0.	If two black holes coalesce, the area of the final event horizon is greater than the sum of the areas of the initial horizons: $A_{+}>A_{1}+A_{2}$ or ∂A/∂t≥0.	You can’t break even.
T3	n,ϵ>0	T>0	κ>0	You have to play.

^1^ The no-hair theorem was posed before black hole mechanics [7].

## 5. Demonstrative Applications

In practice, an observation distribution always has the number of observations *N* as a distribution scaling factor but not necessarily $2^{n}$, as that would require fixing *N* beforehand while the degrees of freedom *n* are typically unknown a priori. Nevertheless, it is obviously the (effective) noise error ϵ within the observations that determines the variance of the Gaussian approximation as $n\epsilon^{2}$, independent of *N*. Moreover, as under-sampling or over-sampling affects all result bins by the same sampling factor, the ratio of the number of observations in the bin of width 2ϵ centered around *m*, denoted $N_{m}$, and the total number of observations *N* (actually within 2nϵ, hence providing a means for outlier screening) are fixed (for odd *n*, the equibinomial distribution actually has two central bins with the same maximum number of observations $N_{m}$; one hence has $2N_{m}$ observations within 4ϵ around *m*, but this can be taken to be $N_{m}$ within 2ϵ as well):

(9) NmN=nn/22n

from Equation (2) with k=n/2. Alternatively, the ratio between the fitting window width 2nϵ and the central bin width 2ϵ equals *n* directly. In combination with $\sigma^{2}=n\epsilon^{2}$ from Gaussian fitting, this allows determining *n* and ϵ for a distribution of repeated observations: One can update the bin width 2ϵ and bin number B=n+1 until $n\epsilon^{2}$ complies with the variance $\sigma^{2}$ from Gaussian fitting. Given an observation distribution, however, one can in principle not distinguish between error contributions originating from the measurand or from the observation system consisting of the measurement equipment, its operator(s), and observer(s). These contributions are dubbed (measurand) variability and (measurement) precision, respectively [11]. Consequently, each observation distribution is expected to combine (at least) two distinct (equi)binomial distributions—here assuming well-defined state variables [12]—although these can often be disentangled in practice, as demonstrated below.

**Fully characterized distribution (precision assessment):** Consider the (repeated) measurement of the length of a rod with a ruler that is longer than the rod. Each length measurement results from two independent readings from the ruler, one at each end of the rod, so n=2 as the number of independent error contributions to the overall assessment. Each reading introduces an error that amounts to half the ruler’s scale $\Delta_{L}$ at maximum, or $\epsilon \equiv \Delta_{L}/2$. The measurement distribution will therefore have three bins of size $\Delta_{L}$ and a variance $\sigma^{2}=n\epsilon^{2}=\Delta_{L}^{2}/2$, irrespective of the amount of measurements made. Now imagine the ruler breaks into two parts that are just shorter than the rod. One hence has to put a mark on the rod, and perform n=4 readings (from the mark to each of the rod’s ends) for each rod length measurement (cf. Figure 1). Although the ensemble’s expectation value and random error remain the same, the new observation distribution will have an increased variance $\sigma^{2}=\Delta_{L}^{2}$. Note that this example neglects other random uncertainty contributions (terms in the second law of thermodynamics), like thermal noise (which here cannot be distinguished between the rod and the ruler), ruler scaling imprecision, reading and recording errors, etc. If the observed variance is significantly larger than just described, one must conclude that these other uncertainty contributions cannot be neglected.

**Archetype statistics (variability assessment):** The Belgian polymath Adolphe Quételet introduced statistical methods to the social sciences in the 19th century. An archetype normal distribution from his hand covers the chest circumference of 5758 “Scottish militiamen” (1846). This distribution consists of B=16 bins, ranging from 33 to 48 inch, that contain 3, 18, 81, 185, 420, 749, 1073, 1079, 954, 658, 370, 92, 50, 21, 4, and 1 count(s), respectively. Fitting a Gaussian function yields $\sigma^{2}=n\epsilon^{2}\approx 4.2$ (square inch), while 2nϵ=15 (inch). Solving this set of equations results in ϵ≈0.56 (inch) and n≈13.4. Taking n≡13 gives ϵ≈0.57 (inch). So by selecting one-inch (2ϵ) bins, Quételet came close to the exact binomial distribution, although it is under-sampled by about 30% (as $1-5758/2^{13}$). Note that if he had selected eight two-inch bins containing 21, 266, 1169, 2152, 1612, 462, 71, and 5 counts, respectively, this under-resolving would indeed have been detected, as Gaussian fitting hence yields ϵ≈0.59 (inch) and n≈12.8, or essentially again n≡13. In any case, for 1 inch result bins, the measurement precision, which is assumed to be of the order of 0.1 inch, can easily be neglected, so the distribution can be considered to be due to ‘natural variability’ only.

**Under-constrained observations (removing precision):** In under-constrained observations, one attempts to retrieve—usually as an ill-posed inverse problem—more measurands about a system than there are independent measurements of that system available. This can only be achieved by making *prior* assumptions about the system that add information to the measurement(s). Each measurand *m* as such is effectively a function F of both the measurement(s) of the true state *t* of the system and the prior assumption(s) *p*: m≡F(t,p), which combines the degrees of freedom *n* and error ϵ originating from the measurement, prior, and retrieval function F. Knowledge of a linear F, e.g., as in remote sounding experiments, enables a *posterior* assessment of the prior-free observation error, originating from the measurement(s) and retrieval only: applying F to an independent reference observation *r* of the system and taking the difference yields a new measurand (i.e., the expectation value of the difference between *m* and the reference as observed by the retrieval system) $m^{\prime }=F\left(t,p\right)-F\left(r,p\right)\equiv F^{\prime }\left(t-r\right)$ that is effectively independent of *p* [13]. The availability of an uncertainty assessment of the reference(s) hence permits determining the degrees of freedom and error of the prior-free observation.

**Comparing observations (removing variability):** Measurements of natural variables most often cannot be *repeated*, think of the atmospheric composition or astronomical events but can be simultaneously (coincident in time, collocated in space) *reproduced* by several instruments instead. Taking the first of two independent observation ensembles as a ‘known’ reference $\left(m_{1},\sigma_{1}\right)$, the bias Δm and variance $\sigma_{2}^{2}=\sigma_{1,2}^{2}-\sigma_{1}^{2}$ of the second can be obtained straightforwardly, with $\sigma_{1,2}^{2}$ the variance of the difference distribution that lacks measurand variability (approximately, cf. the previous case). Once three (or more) independent observation ensembles of multiple system states are available, triple comparisons allow an exact assessment of the measurement variances involved [11]: $\sigma_{1,2}^{2}=\sigma_{1}^{2}+\sigma_{2}^{2}$, $\sigma_{2,3}^{2}=\sigma_{2}^{2}+\sigma_{3}^{2}$, and $\sigma_{3,1}^{2}=\sigma_{3}^{2}+\sigma_{1}^{2}$, resulting in three identical equations for three unknowns, called a three-cornered hat, here in its most simple form. Whenever relevant, however, imperfect coincidences and collocations between the observation systems, called spatiotemporal representativeness differences, have to be taken into account in these expressions as well [14].

**Temperature measurements (as variability or not):** The temperature or variance of a distribution can be a physical measurand under study as such, with $T=2n\epsilon^{2}/k_{B}$. A temperature measurement can therefore be obtained using two distinct approaches: one either performs highly-resolved (kinetic) energy measurements on a system and fits a variance to their distribution, or one calibrates a system that changes with temperature using predefined temperature values. In the first approach, one has to sum the (kinetic) energy of all *n* particles involved, which hence equal the system’s degrees of freedom. The variability or ‘error’ ϵ is determined by the Maxwell–Boltzmann distribution of the particles’ velocities (which actually is a $\chi^{2}$ distribution of three normally distributed variables). The most well-known objectification of the second approach consists of putting a liquid that linearly expands with temperature into a transparent tube, and marking an equidistant scale on the tube between two calibration levels, e.g., of freezing and cooking water (defined as 0 °C and 100 °C, respectively, for the Celsius scale). The measurand as such becomes a scale reading with a distribution shape that is independent of the temperature quantity under study. Rather, the above notes on the fully characterized distribution of ruler readings apply. It is clear that in practice this second approach is preferred, although it critically depends on the preceding calibration operation(s).

## 6. Discussion

We have demonstrated that the common four laws of classical thermodynamics apply to equibinomial observation statistics, with the partition function straightforwardly representing the number of independent observations *N* as the distribution integral. Generalizations, especially of the partition function definition, to quantum-mechanical observed or observation systems should apply, but require more study, both in terms of mathematical rigor and physical interpretation. E.g., for symmetric two-state systems, like a chain of *n* random spins (up or down with equal probability), one expects the above to apply straightforwardly. For systems having more than two states per degree of freedom, the binomial distribution is no longer valid, although a unique temperature—and thermal equilibrium—may still be defined for equally distributed probabilities and equal states ϵ. Think of the Gaussian wave packet that is obtained in the dynamics of large quantum systems (n≫) [15]. Even for two states, whether classical or quantum-mechanical, non-equilibrium distributions originate from shifting state variables *n* or ϵ. Asymmetric or generally non-Gaussian observation distributions, on the other hand, either originate from non-equal states ϵ (resulting in non-equal bin widths) or from non-symmetric state probabilities. In practice, one can think of errors mixing both random and systematic components (unofficially dubbed “headache errors” for obvious reasons [3]), e.g., apart from a constant offset as an additive bias, the ensemble mean *m* can also be shifted by a multiplicative bias component that is equivalent to ϵ∝k.

We have proposed a reformulation of the laws of thermodynamics that summarizes the definition and constraints of equibinomial observation ensembles, matching them with classical thermodynamics terminology. As such, this work provides a direct microscopic interpretation for the entropy, which is different from what has been proposed earlier [16]. Moreover, each term in the first law of thermodynamics is shown to be a variation on $\delta m^{2}=\delta {\left(n\epsilon \right)}^{2}$ for both canonical and macro-canonical observation ensembles, while micro-canonical ensembles correspond to a single observation result bin having $\delta m^{2}=0$. Apart from a scaling factor that equals the partition function, the three state variables *m*, *n*, and ϵ maximally statistically characterize the observation ensemble, and correspond to its expectation value (including systematic error as constant offset), degrees of freedom, and random error, respectively. This is very much in agreement with the recent proposal by the Joint Committee for Guides in Metrology to consider a new definition of measurement uncertainty in the International Vocabulary of Metrology [17]. The new definition includes that “Measurement uncertainty is described fully and quantitatively by a probability distribution on the set of the measurand.” In this work, the state variables and number of observations *N* provide a full quantification of the probability distribution for equibinomial observation distributions. A perfect equibinomial sampling is only obtained for $N\equiv 2^{n}$. Expanding on Ginsberg’s theorem, we could conclude that “with the existence of only few *observable* pieces, nature plays a *hiding* game that you just cannot get out of, and also cannot win”.

## Figures and Tables

**Table 1:** Statistical observation ensembles for the perfect equibinomial distribution with $Z\equiv N=2^{n}$. For different numbers of observations *N*, a correction factor $N/2^{n}$ applies.

Ensemble	Partition Function	Interpretation
Micro-canonical	W(n,k)=Pk=nk	Single bin, ϵ undefined
Canonical	$Z\left(n,\epsilon \right)=\sum_{k}z_{k}\exp\left(-\frac{{\left(2k\epsilon -n\epsilon \right)}^{2}}{2n\epsilon^{2}}\right)$	Sum over equibinomial bins
Macro-canonical	$Z\left(\epsilon \right)=\sum_{k,n}z_{k}\exp\left(-\frac{{\left(2k\epsilon -n\epsilon \right)}^{2}}{2n\epsilon^{2}}\right)$	Sum over equibinomial distributions

## Data Availability

No new data were created or analyzed in this study. Data sharing is not applicable to this article.

## References

[1] Parrondo J.M.R., Horowitz J.M., Sagawa T. (2015). Thermodynamics of Information. Nat. Phys..

[2] Honig J.M. (2021). Thermodynamics: Principles Characterizing Physical and Chemical Processes.

[3] von Clarmann T., Degenstein D.A., Livesey N.J., Bender S., Braverman A., Butz A., Compernolle S., Damadeo R., Dueck S., Eriksson P. (2020). Overview: Estimating and Reporting Uncertainties in Remotely Sensed Atmospheric Composition and Temperature. Atmos. Meas. Tech..

[4] von Clarmann T., Compernolle S., Hase F. (2022). Truth and uncertainty. A critical discussion of the error concept versus the uncertainty concept. Atmos. Meas. Tech..

[5] Ginsberg A. (1975). Article. Coevol. Q..

[6] Bardeen J.M., Carter B., Hawking S.W. (1973). The four laws of black hole mechanics. Commun. Math. Phys..

[7] Israel W. (1967). Event Horizons in Static Vacuum Space-Times. Phys. Rev..

[8] Wang Z.W., Braunstein S.L. (2018). Surfaces away from horizons are not thermodynamic. Nat. Commun..

[9] Keppens A. (2025). Schwarzschild spacetime thermodynamics. SSRN.

[10] Bekenstein J.D. (1974). Generalized second law of thermodynamics in black-hole physics. Phys. Rev. D.

[11] Grubbs F.E. (1948). On estimating precision of measuring instruments and product variability. J. Am. Stat. Assoc..

[12] Korbel J., Wolpert D.H. (2024). Nonequilibrium thermodynamics of uncertain stochastic processes. Phys. Rev. Res..

[13] Keppens A., Compernolle S., Verhoelst T., Hubert D., Lambert J.C. (2019). Harmonization and comparison of vertically resolved atmospheric state observations: Methods, effects, and uncertainty budget. Atmos. Meas. Tech..

[14] Gruber A., Su C.H., Zwieback S., Crow W., Dorigo W., Wagner W. (2016). Recent advances in (soil moisture) triple collocation analysis. Int. J. Appl. Earth Obs. Geoinf..

[15] Cui B. (2023). The classical dynamics for the center of mass of a large quantum system. Phys. Lett. A.

[16] Nojiri S., Odintsov S.D., Paul T. (2023). Microscopic interpretation of generalized entropy. Phys. Lett. B.

[17] Possolo A. (2025). Measurement uncertainty redefined. Metrologia.

